# Hyperphosphorylation of RPS6KB1, rather than overexpression, predicts worse prognosis in non-small cell lung cancer patients

**DOI:** 10.1371/journal.pone.0182891

**Published:** 2017-08-09

**Authors:** Bojiang Chen, Lan Yang, Rui Zhang, Yuncui Gan, Wen Zhang, Dan Liu, Hong Chen, Huairong Tang

**Affiliations:** 1 Department of Respiratory and Critical Care Medicine, West China Hospital of Sichuan University, Chengdu, China; 2 Department of General Medicine, Sichuan Academy of Medical Sciences & Sichuan Provincial People's Hospital, Chengdu, China; 3 Health Management Center, West China Hospital, Sichuan University Chengdu, Sichuan, China; University of North Carolina at Chapel Hill School of Medicine, UNITED STATES

## Abstract

RPS6KB1 is the kinase of ribosomal protein S6 which is 70 kDa and is required for protein translation. Although the abnormal activation of RPS6KB1 has been found in types of diseases, its role and clinical significance in non-small cell lung cancer (NSCLC) has not been fully investigated. In this study, we identified that RPS6KB1 was over-phosphorylated (p-RPS6KB1) in NSCLC and it was an independent unfavorable prognostic marker for NSCLC patients. In spite of the frequent expression of total RPS6KB1 and p-RPS6KB1 in NSCLC specimens by immunohistochemical staining (IHC), only p-RPS6KB1 was associated with the clinicopathologic characteristics of NSCLC subjects. Kaplan-Meier survival analysis revealed that the increased expression of p-RPS6KB1 indicated a poorer 5-year overall survival (OS) for NSCLC patients, while the difference between the positive or negative RPS6KB1 group was not significant. Univariate and multivariate Cox regression analysis was then used to confirm the independent prognostic value of p-RPS6KB1. To illustrate the underlying mechanism of RPS6KB1 phosphorylation in NSCLC, LY2584702 was employed to inhibit the RPS6KB1 phosphorylation specifically both in lung adenocarcinoma cell line A549 and squamous cell carcinoma cell line SK-MES-1. As expected, RPS6KB1 dephosphorylation remarkably suppressed cells proliferation in CCK-8 test, and promoted more cells arresting in G0-G1 phase by cell cycle analysis. Moreover, apoptotic A549 cells with RPS6KB1 dephosphorylation increased dramatically, with an elevating trend in SK-MES-1, indicating a potential involvement of RPS6KB1 phosphorylation in inducing apoptosis. In conclusion, our data suggest that RPS6KB1 is over-activated as p-RPS6KB1 in NSCLC, rather than just the total protein overexpressing. The phosphorylation level of RPS6KB1 might be used as a novel prognostic marker for NSCLC patients.

## Introduction

Lung cancer is one of the most prevalent malignancies in both China and worldwide [1]. Despite the advances in surgical techniques, radiotherapy and targeted drugs, the prognosis of lung cancer patients has not improved, with a 5-year overall survival (OS) rate of 15% [1, 2]. It has been widely accepted that the unclear pathological mechanism is the primary barrier to lung cancer intervention. Novel biomarkers are urgently required to predict the patients clinicopathological and prognostic features.

Proteins are functional executors of genes. Ribosomal protein S6 kinase beta-1 (*RPS6KB1*) encodes the 70 kDa ribosomal protein S6 kinase (RPS6KB1), which is also known as p70 S6 kinase (p70S6K) [3]. RPS6KB1 is a serine/threonine kinase regulated by phosphoinositide 3-kinase (PI3K)/mammalian target of rapamycin (mTOR) pathway, and activates the substrate ribosomal protein S6 to induce protein synthesis by translating the 5’-terminal oligopyrimidine tract mRNAs [3, 4]. Increasing evidence suggests that RPS6KB1 is involved in several types of diseases and normal physiological processes [5–7]. During the soleus muscle lengthening and growing, RPS6KB1 is phosphorylated by passive stretch and promotes protein translation [8]. In a high fat diet mice model, absence or knockdown of RPS6KB1 impaired the early adipocyte progenitor generation and attenuated the adipogenesis, indicating a critical role of RPS6KB1 in obesity [9]. RPS6KB1 was also found to be involved in the KCl withdrawal-induced cerebellar granule neuron apoptosis [10]. The Additionally, overexpression of RPS6KB1 was observed in breast cancer cells as well [11], and greatly promoted the cells proliferation [12]. However, the role of RPS6KB1 was poorly understood. Since more than 80% of lung cancer patients are diagnosed as non-small cell lung cancer (NSCLC) according to the pathological characteristics, the current exploration was carried out in NSCLC. Firstly, the prognostic value of RPS6KB1 in NSCLC patients was investigated. Results revealed that both RPS6KB1 and its phosphorylated protein p-RPS6KB1 at T389 were highly up-regulated in NSCLC tissues, compared with controls; but only the hyperphosphorylation independently predicted the adverse prognosis of NSCLC patients. Subsequent dephosphorylation of RPS6KB1 by specific inhibitor evidently reduced the NSCLC cells proliferation via inducing G0-G1 cell cycle arrest. Our study showed the crucial role of RPS6KB1 in NSCLC preliminarily.

## Materials and methods

### Patients and specimens collection

This study was approved by the Clinical Research Ethics Board of West China Hospital of Sichuan University, China [13]. Two hundred and thirty-eight cases who received the complete resection for primary NSCLC in West China Hospital from 2010 to 2011 were recruited retrospectively. However, only 160 subjects with full clinical information from the medical record, prognostic data of overall survival (OS) by telephone follow-up and the consent to participating in the study were finally enrolled. OS, the prognostic index in this study, was defined as time from operation to death or the status at the last follow-up[14]. None of the patients underwent previous neoadjuvant therapy, and all subjects received standard therapeutic procedures after surgical resection according to the National Comprehensive Cancer Network (NCCN) guidelines of NSCLC. The archived resected cancerous tissues and part of paired adjacent normal controls over 5 cm away from tumors (86 cases) were collected in the Department of Pathology. All specimens were fixed in 10% formalin immediately and embedded in paraffin within 24 h after the surgical resection. The pathologic diagnosis, as well as tumor differentiation and histological subtypes, was made by two experienced pathologists in double blind, according to the 2015 WHO classification of lung tumors [15]. The TNM stages were classified in the light of the Eighth Edition of International Association for the Study of Lung Cancer (IASLC) International Staging Project [16], since we conducted the data analysis in the second half of 2016. Moreover, even though with consent from patients and ethical approval from the Ethics Board, data were still fully anonymized prior to accessing by researchers.

### Immunohistochemical (IHC) staining

All paraffin-embedded tissues were sectioned at 4 μm in thickness for IHC staining, and the procedure was carried out as the manual of EnVision^™^ Detection Systems Peroxidase/DAB (Dako, Denmark, #K5007). The epitope retrieval was performed by heat-induced technique. Briefly, tissue slides were deparaffinized in gradient xylene and ethanol, and then boiled in a pressure cooker with 0.01 M citrate buffer (pH 6.0) for 10 minutes. After cooling down to the room temperature, sections were treated by serum blocking reagent for 30 min. Primary antibody was then used for incubation at 4°C in a humidity chamber overnight, followed by the secondary antibody, DAB developer and hematoxylin staining application in turn. Commercially purchased IHC primary antibodies directed against the following antigens were: RPS6KB1 (Anti-RPS6KB1 E343 antibody, #ab32529), p-RPS6KB1 (Anti-RPS6KB1 phospho T389 antibody, #ab126818). The confirmed colon carcinoma specimens were used for positive controls, while PBS instead of the primary antibodies were negative controls [17].

### Evaluation of IHC staining

The IHC staining was evaluated blindly by two pathologists in a semiquantitative method, without any knowledge of the patients’ clinical information. Ten areas were selected randomly under light microscopy in each slide, and scored for both of the intensity and quantity of positively stained cells. The intensity was referred to as: 0 for no appreciable staining; 1 for barely detectable staining; 2 for readily appreciable brown staining and 3 for dark brown staining. Whereas the quantity was the percentage of positive cells, scored as 0 for no staining; 1 for < 20% cells stained; 2 for 20% - 50% cells stained; and 3 for > 50% cells stained. The total score was calculated by multiplying the intensity with the quantity scores, ranging from 0 to 9. For statistical analysis, IHC staining was categorized as negative (0–2 scores) and positive (3–9 scores) [13].

### Cell culture and drug treatment

Human lung adenocarcinoma cell line A549 and squamous cell carcinoma cell line SK-MES-1 were obtained from ATCC, and cultured according to the supplier’s instructions. Briefly, cell lines were cultured a humidified incubator containing 5% CO_2_ and 95% ambient air at 37°C by RPMI-1640 medium supplemented with 10% fetal bovine serum and 1% penicillin/streptomycin [18].

LY2584702 was provided by Selleck Chemicals (USA, #S7698) with solubility in warmed DMSO of 1 mg/mL (2.24 mM) and IC50 of 4 nM. One mg of LY2584702 were fully dissolved in 20 ml 10% DMSO and reserved at -80°C. When conducted the experiments *in vitro*, LY2584702 was further diluted in 0.5% Tween 80, 5% propylene glycol and 30% PEG400 to reach different DMSO concentrations of 0.1 μM, 0.2 μM, 0.6 μM, and 1.0 μM.

### Western blot analysis

Cell lines treated with LY2584702 for 24 h at different concentrations were harvested. Total or phosphorylated protein was isolated using the Total Protein Extraction Kit (KeyGEN, Nanjing, China, #KGP2100) or Phosphorylated Protein Extraction Kit (KeyGEN, Nanjing, China, #KGP9100), respectively. Then protein concentrations were measured by BCA Protein Assay Reagent (Thermo Scientific, Rockford, USA, #23223). After separating on polyacrylamide gels (Bio-Rad, Richmond, CA) by electrophoresis, proteins were transferred to blot membranes. Blots were then incubated with the primary antibodies against RPS6KB1 (Anti-RPS6KB1 E343 antibody, #ab32529), p-RPS6KB1 (Anti-RPS6KB1 phospho T389 antibody, #ab126818), S6 (Anti-S6, #ab40820), p-S6 (Anti-S6, phospho S235/S236, #ab12864) and β-actin (Controls, Anti-β-actin, #ab8226), which were followed by secondary antibodies conjugated to HRP. Enhanced chemiluminescence substrate (Thermo-Fisher Scientific, Waltham, MA) were added up to blots for image acquisition and the band density was analyzed with an automated imager (Protein Simple, Santa Clara, CA)[19].

### Cell proliferation assay

Cell Counting Kit-8 (CCK-8; Dojindo Laboratories, Kumamoto, Japan) was used to measure the cells proliferation *in vitro*. Cells treated by LY2584702 for 24 h with different concentrations were seeded in 96-well plates at a density of 5×10^3^ per well, with six repeats. DMSO treated, or in other words, the concentration of LY2584702 of 0 was used as negative control. Cells absorbance at 450 nm was detected every 24 h after seeding to measure the proliferative activities [20].

### Cell cycle analysis

Based on the results of CCK-8 test, A549 treated by LY2584702 at 0.2 μM and SK-MES-1 treated at 1 μM for 72 h were harvested for cell cycle and apoptosis analysis. Cells were rinsed by cold PBS two times, and fixed in 80% ice-cold ethanol for at least 4 h, followed by propidium iodide/RNase staining buffer suspending (BD Pharmingen, USA, #550825). DNA contents were detected by a FACSCalibur Flow Cytometer (BD, Franklin Lakes, USA) as an index of cell cycle distribution. Cells were divided into G0-G1, S and G2-M phases and data were analyzed using the CellQuest and Modfit software. Experiments were repeated three times independently [21].

### Cell apoptosis analysis

Cells were digested by trypsin without EDTA. Flow cytometry (BD FACSCalibur, Becton, USA) was used to detect the apoptotic cells after full staining by Annexin V-APC/7-AAD Apoptosis Detection Kit (KeyGen, Nanjing, China). According to the manufacturer’s protocol, cells with positive Annexin V staining and negative 7AAD staining were regarded as apoptotic. Experiments were repeated three times independently [22].

### Statistical analysis

The difference between the expression of RPS6KB1 and p-RPS6KB1 in clinical tissues, and the correlation between RPS6KB1 and p-RPS6KB1 expression and clinicopathologic variables, were evaluated by Chi-square test. Kaplan-Meier method with *Log Rank* test was used to estimate the patients’ 5-year OS with different RPS6KB1 and p-RPS6KB1 expression, or with different clinical characteristics. The patients’ status was censored if lost to follow-up. Univariate and multivariate Cox proportional hazards regression models were employed to determine the significant prognostic factors and calculate their hazard ratios (HRs) with 95% confidential intervals (*CI*s). Data in the proliferation, cell cycle and apoptosis were expressed as mean ± Standard Deviation (SD), and one way-ANOVA with *LSD* test was applied for analysis. All statistical analysis was performed using SPSS software, version 19.0 (SPSS Inc, Chicago, IL). *P* value less than 0.05 was considered as statistically significant.

## Results

### Total RPS6KB1 and p-RPS6KB1 are both overexpressed in NSCLC patients

**Fig 1:**
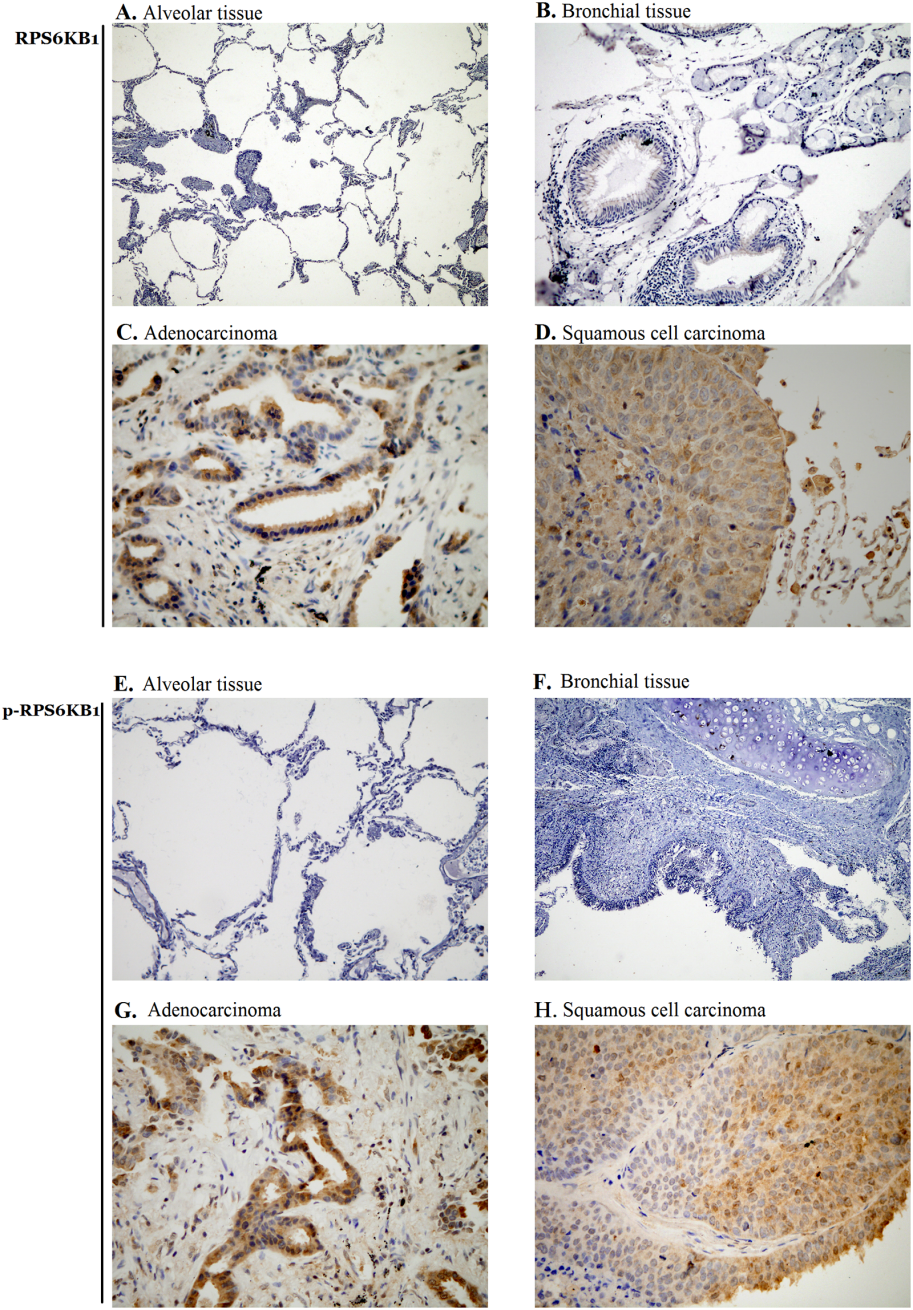
Expression of RPS6KB1 and p-RPS6KB1 NSCLC samples and controls. (A, B) Representative images of negative expression of total RPS6KB1 in normal alveolar tissue and bronchial tissue (× 100). (C, D) Representative images of strong expression of total RPS6KB1 in lung adenocarcinoma and squamous cell carcinoma (× 200). (E, F) Representative images of negative expression of p-RPS6KB1 in normal alveolar tissue and bronchial tissue (× 100). (G, F) Representative images of strong expression of p-RPS6KB1 in lung adenocarcinoma and squamous cell carcinoma (× 200).

IHC staining was conducted on 160 surgical NSCLC samples and 86 corresponding adjacent non-cancerous tissues. As shown in Fig 1, total RPS6KB1 and p-RPS6KB1 were both mainly detected in cytoplasm. Staining scores for RPS6KB1 and p-RPS6KB1 expression in tumor tissues were between 0 and 9, with 81.25% (130/160) and 61.25% (98/160) cases divided into the positive group (no less than 3 scores). Whereas, control biopsies received scores for RPS6KB1 and p-RPS6KB1 of 0 to 6 with none of them achieving either 7 to 9, and the positive rates decreased to 58.14% (50/86) and 41.86% (36/86), respectively. Chi-square analysis revealed the significantly higher positive expression of total RPS6KB1 and p-RPS6KB1 in NSCLC tissues than those in controls (Table 1, *P* < 0.001 and = 0.004, respectively), indicating a potential key role of RPS6KB1 in NSCLC.

**Table 1:** Expression of RPS6KB1 and p-RPS6KB1 in NSCLC and normal lung tissues (IHC staining).

Proteins	Expression	NSCLC tissues (*n*, %)	Normal lung tissues (*n*, %)	*P*
**RPS6KB1**	P	130 (81.25)	50 (58.14)	< 0.001*
	N	30 (18.75)	36 (41.86)	
**p-RPS6KB1**	P	98 (61.25)	36 (41.86)	0.004*
	N	62 (38.75)	50 (58.14)	

P: Positive expression; N: Negative expression.

*: *P* < 0.05.

### Clinicopathologic significance of RPS6KB1 and p-RPS6KB1 in NSCLC patients

The relationship between total RPS6KB1, p-RPS6KB1 and clinical variables was summarized in Table 2. Overexpression of RPS6KB1 did not correlate with any of the demographic or clinicopathologic characteristics (all *P* > 0.05). However, the high expression of p-RPS6KB1 was more frequent in regional lymph node involved or advanced stage cases (Table 2, *P* = 0.033 and < 0.001, respectively), though there was no association between p-RPS6KB1 overexpression and gender, age, histological type, differentiation, tumor size or distant metastasis (all *P* > 0.05). The results suggested that RPS6KB1 might be hyperphosphorylated to exert its pathological functions in NSCLC.

**Table 2:** Relationship between clinical characteristics and RPS6KB1 & p-RPS6KB1 expression.

Factors		Positive expression of RPS6KB1		Positive expression of p-RPS6KB1	
		*n* (%)	*P*	*n* (%)	*P*
**Gender**	Male (*n* = 126)	102 (80.96)	0.853	78 (61.90)	0.743
	Female (*n* = 34)	28 (82.35)		20 (58.82)	
**Age/years**	<60 (*n* = 70)	54 (77.14)	0.240	40 (57.14)	0.347
	≥60 (*n* = 90)	76 (84.44)		58 (64.44)	
**Histological Type**	ADC (*n* = 74)	64 (86.49)	0.055	40 (54.05)	0.079
	SCC (*n* = 64)	52 (81.25)		46 (71.88)	
	Other (*n* = 22)	14 (63.64)		12 (54.55)	
**Histological Differentiation**	Poor (*n* = 64)	54 (84.38)	0.408	40 (62.50)	0.296
	Moderate/Well (*n* = 96)	76 (79.17)		52 (54.17)	
**Tumor Size/cm**	T1 (*n* = 48)	38 (79.17)	0.249	30 (62.50)	0.528
	T2 + T3 + T4 (*n* = 112)	77 (68.75)		64 (57.14)	
**Regional lymph node involvement**	N0 (*n* = 76)	60 (78.85)	0.478	40 (52.63)	0.033*
	N1 + N2 + N3 (*n* = 84)	75 (83.33)		58 (69.05)	
**Distant Metastasis**	M0 (*n* = 150)	124 (82.67)	0.093	92 (61.33)	1.000
	M1 (*n* = 10)	6 (60.00)		6 (60.00)	
**Stage**	I (*n* = 50)	40 (80.00)	0.785	16 (32.00)	< 0.001*
	II+III+IV (*n* = 110)	90 (81.82)		82 (74.55)	

ADC, adenocarcinoma; SCC, squamous cell carcinoma.

*: *P* < 0.05

### Hyperphosphorylation, rather than overexpression, of RPS6KB1 predicts worse 5-year overall survival (OS) for NSCLC patients

**Fig 2:**
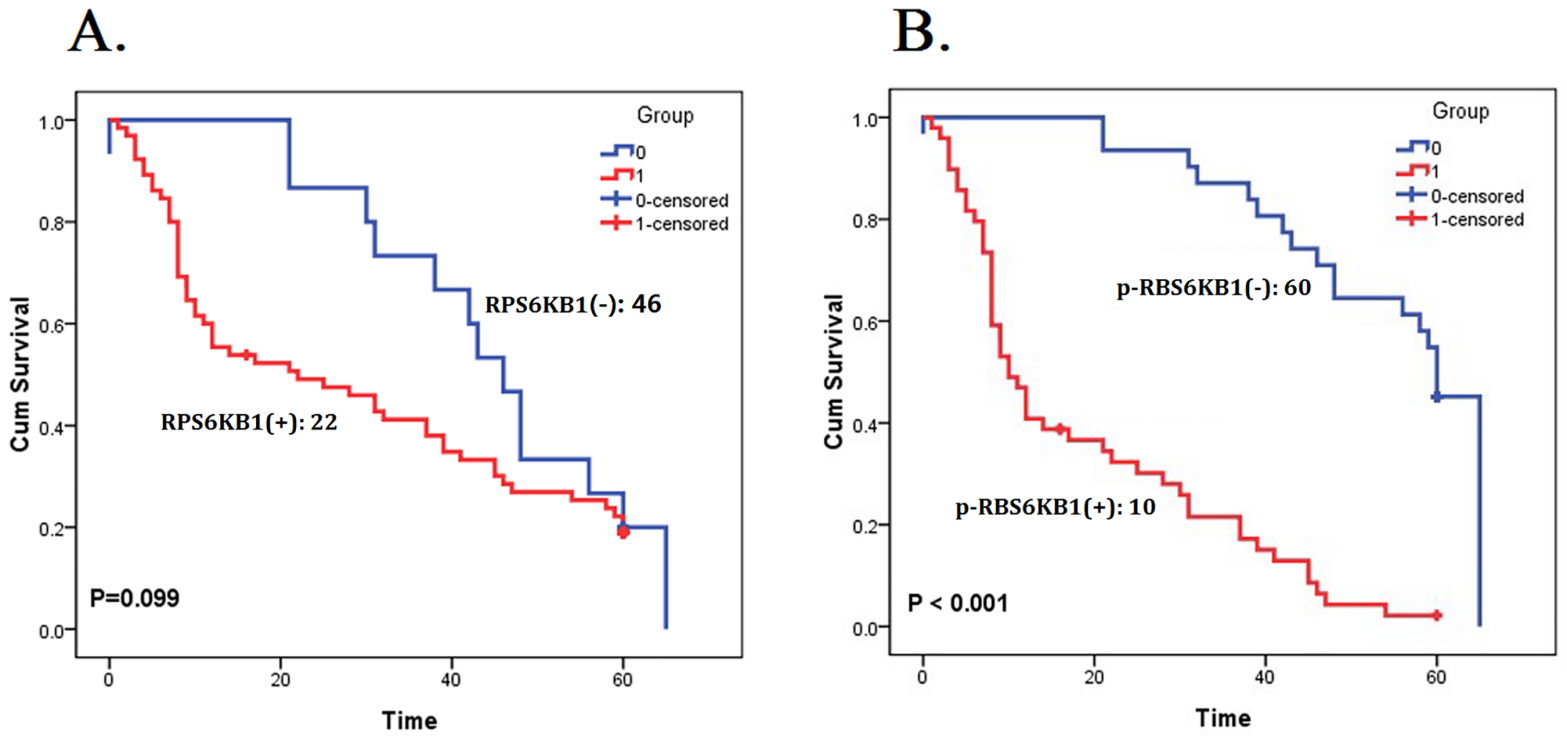
Expression of RPS6KB1 and p-RPS6KB1 in tumor tissues and the 5-year overall survival (OS) of NSCLC patients. Kaplan-Meier survival analyzed the correlation between RPS6KB1 expression (A) and p-RPS6KB1 expression (B) and the 5-year OS of NSCLC patients, who were classified as negative expression (Blue) or positive expression (Red). (*Log Rank* test).

Kaplan-Meier analysis was then used to explore the association between the expression of RPS6K1 and the 5-year OS of NSCLC patients. Results showed that patients with positive expression of RPS6KB1 seemed to have a worse prognosis than negative ones, but the difference did not achieve a significance (Table 3, Fig 2A, *P* = 0.099, *Log Rank* test). However, subjects whose samples were positive for p-RPS6KB1 had a markedly unfavorable survival (Table 3, Fig 2B, *P* < 0.001, *Log Rank* test), suggesting the hyperphosphorylation of RPS6KB1 in NSCLC, rather than only overexpression.

**Table 3:** Relationship between RPS6KB1 & p-RPS6KB1 expression and survival time.

Proteins	Expression	*n*	Median survival time (months)	*Log rank P*
**RPS6KB1**	P	130	22	0.099
	N	30	46	
**p-RPS6KB1**	P	98	10	< 0.001*
	N	62	60	

P: Positive expression; N: Negative expression.

*: *P* < 0.05.

**Fig 3:**
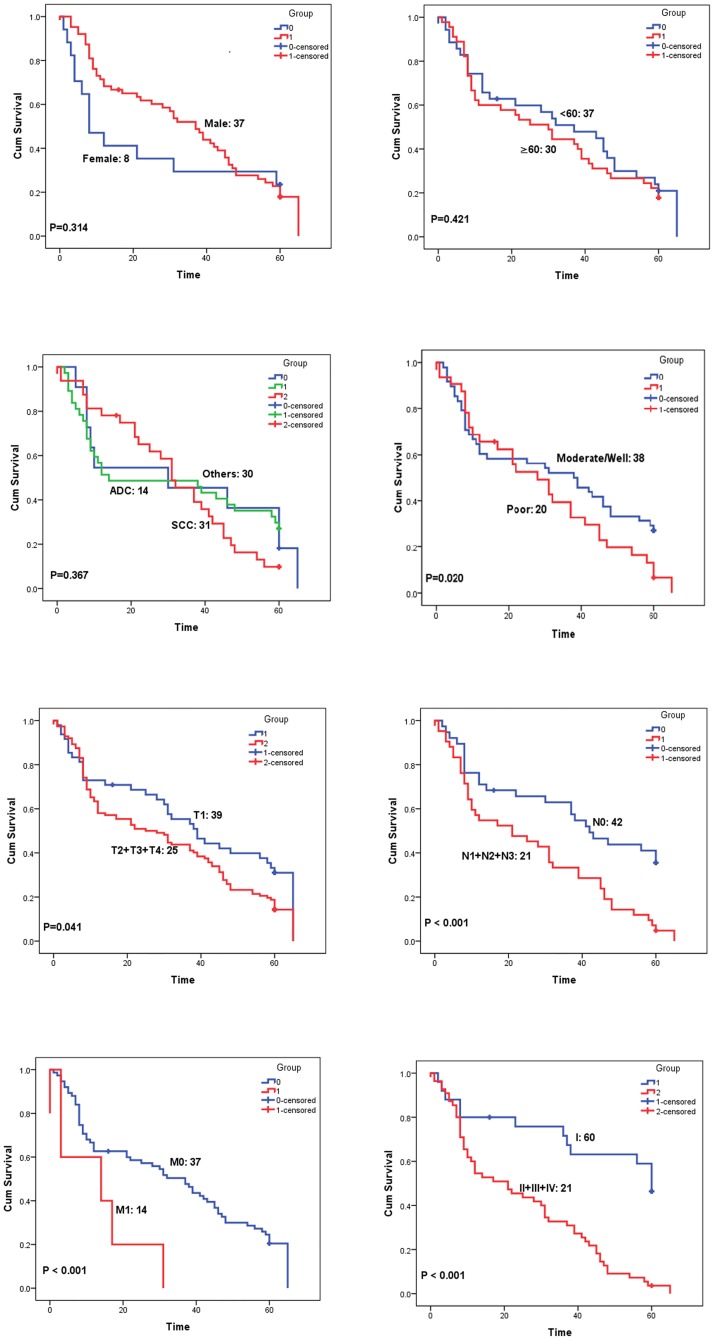
Clinical characteristics and the 5-year overall survival (OS) of NSCLC patients. Kaplan-Meier survival analyzed the correlation between the clinical characteristics and 5-year OS of NSCLC patients. The figures showed gender, age, tumor histological type, histological differentiation, tumor size, regional lymph node involved, distant metastasis, and clinical stage in order. (*Log Rank* test).

Poor histological differentiation, tumor size over 3 cm, regional lymph node involved and distant metastasis were all also shown to associate with unfavorable prognosis of NSCLC patients (Table 4, Fig 3, all *P* < 0.05).

**Table 4:** Relationship between clinical characteristics and survival time (*Log Rank*).

Factors		*n*	Median survival time (months)	*Log rank P*
**Gender**	Male	126	37	0.314
	Femal	34	8	
**Age/years**	<60	70	37	0.421
	≥60	90	30	
**Histological Type**	ADC	74	14	0.367
	SCC	64	31	
	Other	22	30	
**Histological Differentiation**	Poor	64	28	0.020*
	Moderate/Well	96	38	
**Tumor Size/cm**	T1	48	39	0.041*
	T2 + T3 + T4	112	25	
**Regional lymph node involvement**	N0	76	42	< 0.001*
	N1 + N2 + N3	84	21	
**Distant Metastasis**	M0	150	37	< 0.001*
	M1	10	14	
**Stage**	I	50	60	< 0.001*
	II + III + IV	110	21	

ADC, adenocarcinoma; SCC, squamous cell carcinoma.

*: *P* < 0.05

### Hyperphosphorylation of RPS6KB1 is an independent prognostic factor for the 5-year overall survival (OS) in NSCLC patients

Univariate Cox proportional hazards regression analysis was then employed to explore the clinicopathological significance of RPS6KB1, and the differentiation, tumor size, regional lymph node involved, distant metastasis, clinical advanced stage and the expression of p-RPS6KB1 were found to be significant prognostic factors (Table 5, all *P* < 0.05).

**Table 5:** Relationship between clinical characteristics, RPS6KB1 & p-RPS6KB1 expression and survival time (Univariate analysis).

Factors		*n*	Median survival time (month)	HR	95% *CI*	*P*
**Histological Differentiation**	Poor	64	28	1.501	1.056–2.134	0.024*
	Moderate/Well	96	38			
**Tumor Size/cm**	T1	48	39	1.488	1.001–2.212	0.049*
	T2+T3+T4	112	25			
**Regional lymph node involvement**	N0	76	42	2.299	1.595–3.315	< 0.001*
	N1+N2+N3	84	21			
**Distant Metastasis**	M0	150	37	3.098	1.594–6.021	0.001*
	M1	10	14			
**Stage**	I	50	60	4.007	2.538–6.327	< 0.001*
	II+III+IV	110	21			
**p-RPS6KB1**	P	98	10	6.893	4.440–10.701	< 0.001*
	N	62	60			

ADC, adenocarcinoma; SCC, squamous cell carcinoma.

*: *P* < 0.05.

Further multivariate Cox regression analysis showed the stong expression of p-RPS6KB1, together with advanced stage, was an independent prognostic parameter (Table 6, HR = 3.228 and 2.377 respectively, both *P* < 0.05). Thus, hyperphosphorylation of RPS6KB1 was confirmed as an important factor in predicting the OS of NSCLC patients.

**Table 6:** elationship between clinical characteristics, RPS6KB1 & p-RPS6KB1 expression and survival time (Multivariate Cox regression model with the Method of Forward *LR*).

Factors		HR	95%*CI*	*P*
**Stage**	I *vs* II+III+IV	2.377	1.285–4.492	0.008*
**p-RPS6KB1**	P *vs* N	3.228	1.891–5.508	0.005*

P: Positive expression; N: Negative expression.

*: *P* < 0.05.

### The inhibitor of RPS6KB1, LY2584702, significantly reduced the phosphorylation of RPS6KB1 and rpS6 in NSCLC cell lines

**Fig 4:**
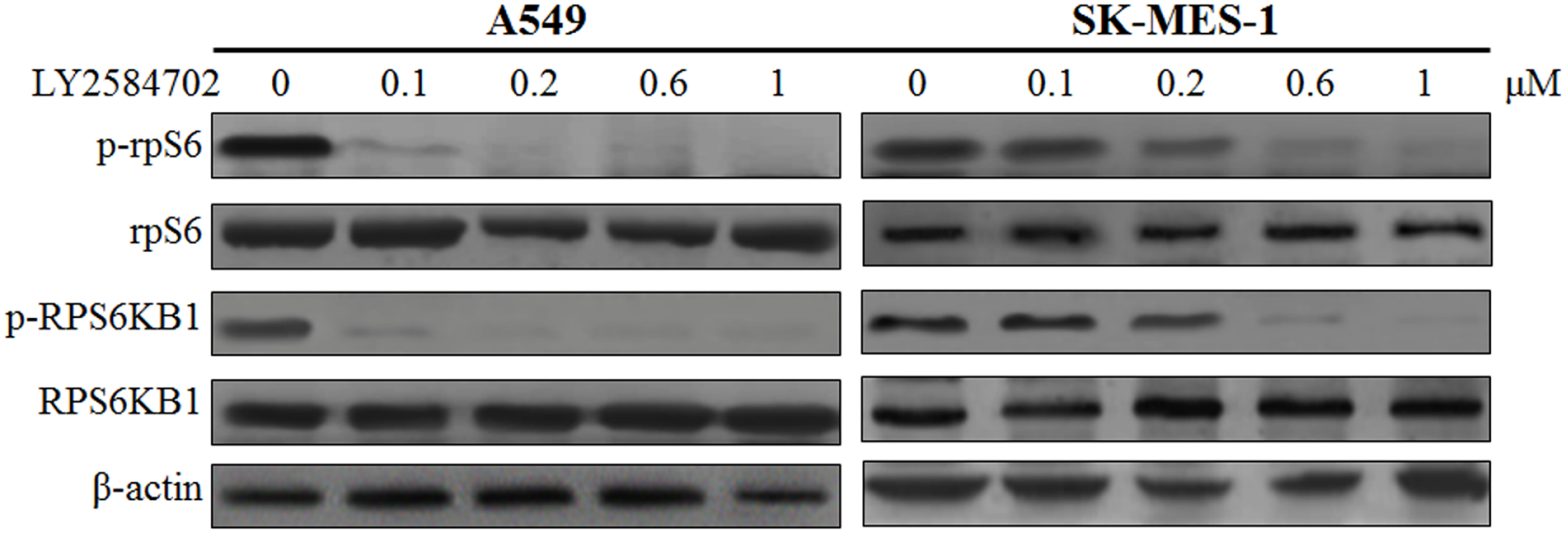
Protein expression of RPS6KB1, p-RPS6KB1, rpS6 and p-rpS6 after the treatment by various LY2584702 concentrations for 24 h. Western blot analysis of RPS6KB1, p-RPS6KB1, rpS6 and p-rpS6 in adenocarcinoma cell line A549 and squamous cell carcinoma cell line SK-MES-1 with various LY2584702 concentrations. DMSO without any LY2584702 were expressed as a concentration of 0. β-actin was used as a loading control.

LY2584702 was used to inhibit the phosphorylation of RPS6KB1 in pulmonary adenocarcinoma cell line A549 and squamous cell carcinoma cell line SK-MES-1. As expected, after the treatment for 24 h, phosphorylation of RPS6KB1 in A549 was markedly reduced even at 0.1 μM (Fig 4, *P* < 0.001); while the expression of p-RPS6KB1 in SK-MES-1 seemed to start decreasing at 0.2 μM and showed a continous down-regulation with increased drug concentrations (Fig 4, all *P* < 0.05). The phosphorylation of rpS6, generally accepted target of RPS6KB1, was also synchronously declined (Fig 4, all *P* < 0.05). However, there was no significant difference in total protein level of neither RPS6KB1 nor S6, no matter with the drug concentration (Fig 4, all *P* > 0.05).

### The dephosphorylation of RPS6KB1 greatly inhibited NSCLC cells proliferation, promoted G0-G1 cell cycle arrest and partly induced cell apoptosis

**Fig 5:**
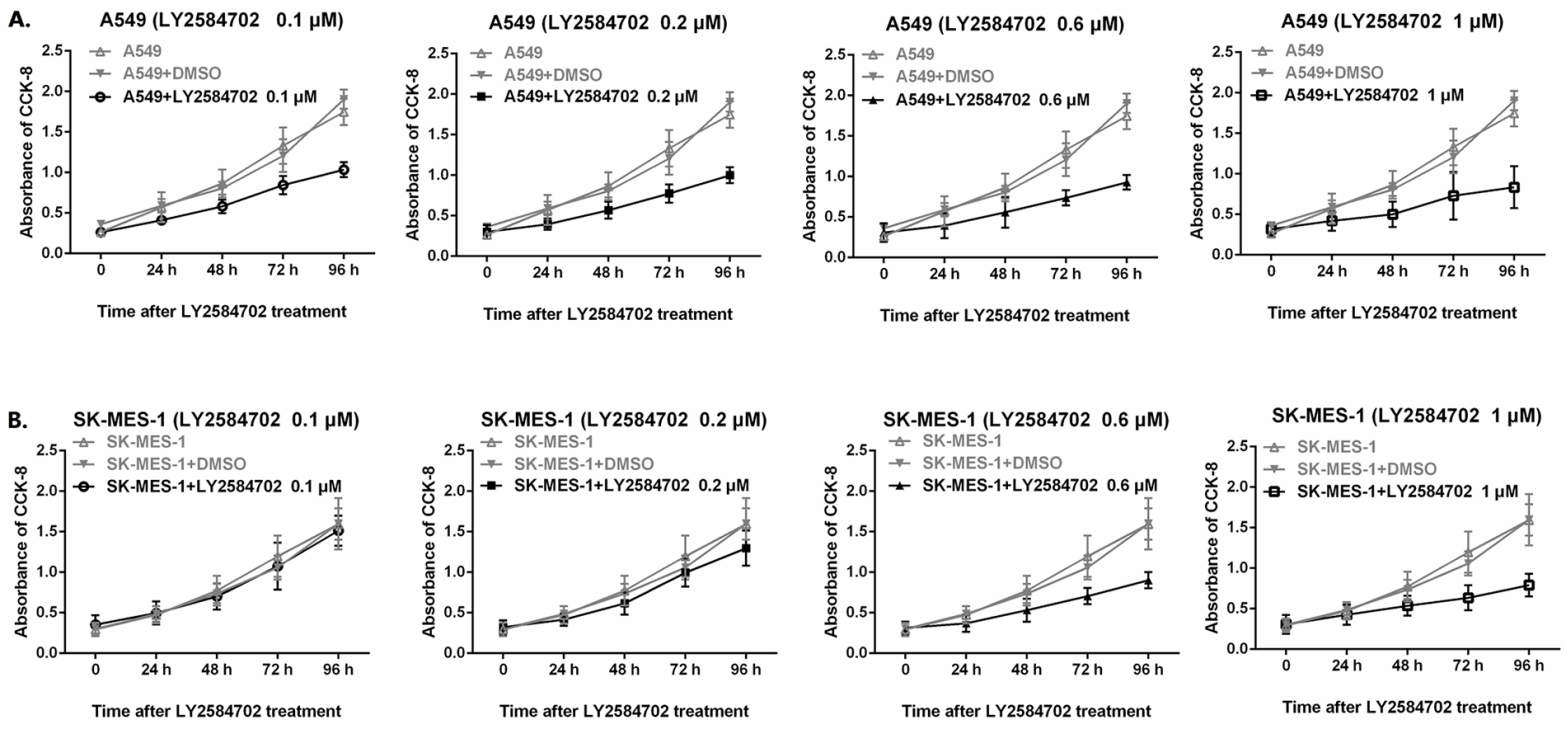
Proliferation alteration of NSCLC cell lines with RPS6KB1 dephosphorylation by LY2584702 (CCK-8 analysis). Cell lines A549 (A) and SK-MES-1 (B) with different LY2584702 concentrations treatment were detected by CCK-8 kit for the proliferation alterations. Cell lines with regular culture or culture medium with DMSO were controls. The obvious inhibition in A549 was observed at LY2584702 0.1 μM for 24 h (*P* = 0.035 and 0.02 when compared with A549 in regular culture or culture medium with DMSO, respectively); while in SK-MES-1, the change became detectable at 0.6 μM for 24 h (*P* = 0.042 and 0.036 when compared with regular culture or culture medium with DMSO, respectively). Both of A549 and SK-MES-1 showed significant time- and concentration-dependence. Data were repeated six times independently.

Proliferation of A549 was significantly inhibited by LY2584702 treating over 24 h at 0.1 μM (Fig 5A, *P* < 0.05); and the trend of decline was more conspicuous with longer treatment and/or with the increased drug concentration (Fig 5A, all *P* < 0.05). Similar results were also observed in SK-MES-1, although the obvious inhibition was led by LY2584702 at 0.6 μM (Fig 5B, all *P* < 0.05), much higher than that of A549.

**Fig 6:**
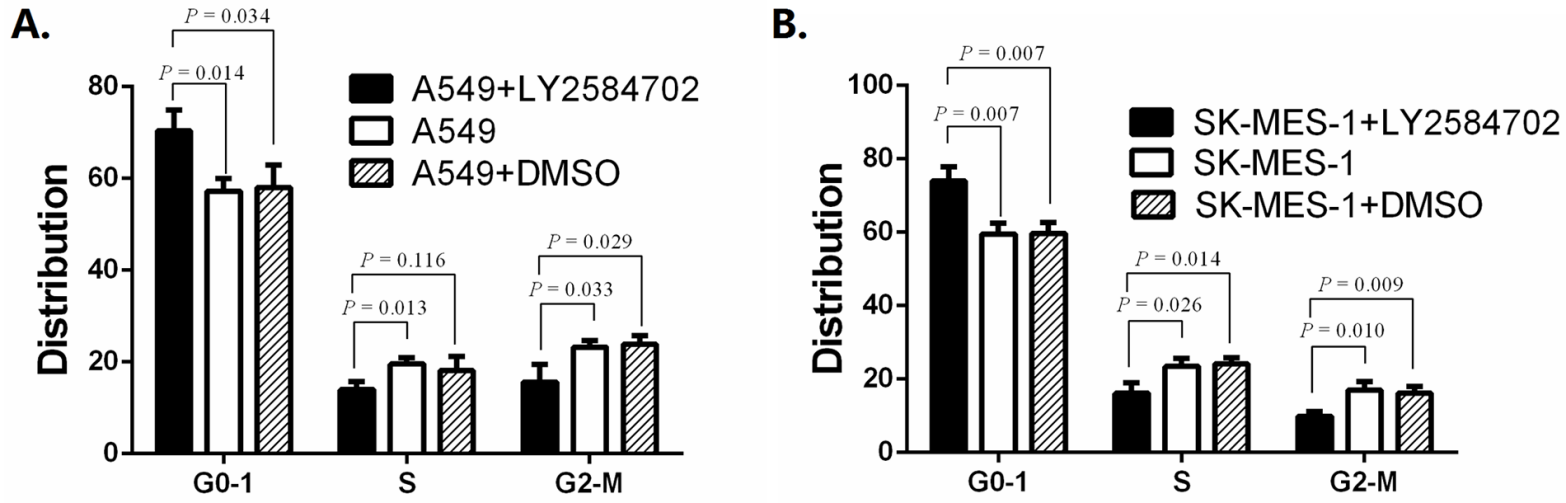
Cell cycle distribution of NSCLC cell lines with RPS6KB1 dephosphorylation by LY2584702. Cell lines A549 (A) treated by LY2584702 at 0.2 μM for 72 h were collected for cell cycle distribution detection and showed G0-G1 arrest (*P* < 0.05). SK-MES-1 (B) were treated by LY2584702 at 1 μM for 72 h and also revealed G0-G1 accumulation (*P* < 0.05). Experiments were independently repeated three times.

**Fig 7:**
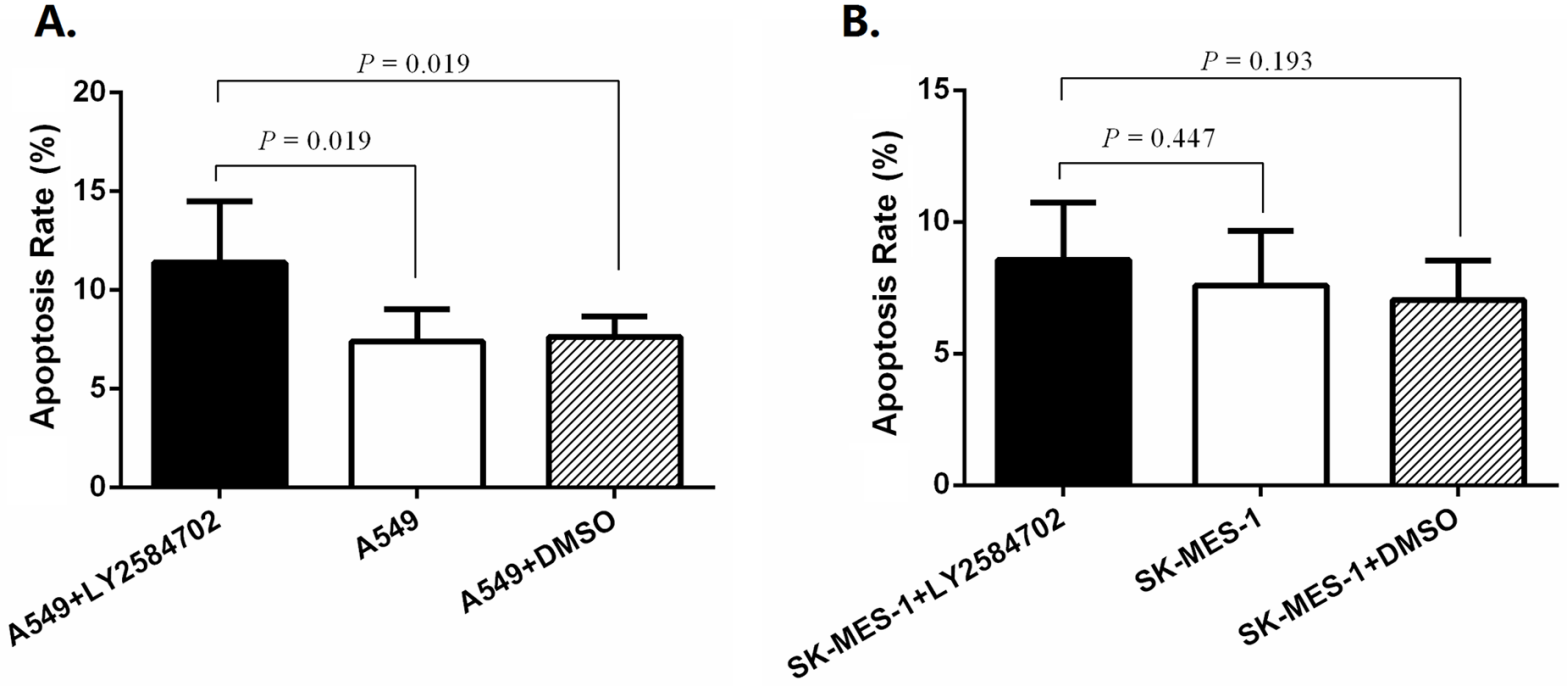
Apoptosis of NSCLC cell lines with RPS6KB1 dephosphorylation by LY2584702. Cell lines were also collected to detect apoptosis. The apoptotic ratio of A549 (A) increased significantly by LY2584702 at 0.2 μM for 72 h (*P* < 0.05). However, the elevated difference in SK-MES-1 (B) did not reach a statistical significance (*P* > 0.05). Experiments were independently repeated three times.

Based on the results above, A549 treated by LY2584702 at 0.2 μM for 72 h were collected for cell cycle assay and apoptosis analysis. A549 cell lines cultured only by medium or medium added with DMSO for 72 h were used as controls. Not surprisingly, more cells with LY2584702 treatment were arrested in G0-G1 phase (Fig 6A, both *P* < 0.05); and cells in S or G2-M phase decreased correspondingly (Fig 6A, both *P* < 0.05). In addition, LY2584702 induced more apoptotic A549 cell by Annexin V-APC/7-AAD apoptosis detection (Fig 7A, both *P* < 0.05).

Because of the less sensitivtiy of SK-MES-1 for LY2584702, SK-MES-1 treated at 1 μM for 72 h were employed for the cell cycle and apoptosis analysis. Silimarly, compared with controls, LY2584702 treatment also led to SK-MES-1 G0-G1 arrest and synchronous reduction in S and G2-M phase (Fig 6B, all *P* < 0.05). However, LY2584702 showed a limited effect on SK-MES-1 apoptosis, in spite of a vague increase trend (Fig 7B, both *P* > 0.05).

## Discussion

Lung cancer is highly heterogeneous. It is difficult to identify patients at greatest risk for malignant progression, which is conditioned by different biological factors that regulate cell proliferation, angiogenesis, apoptosis, and so on. Some of these factors have been evaluated to find helpful tools to predict the patients’ prognosis, but results have been so far inconclusive [23]. Compared with gene detection, relatively expensive and not widely available, protein expression measurement, such as IHC staining, has been routinely carried out in most hospitals, and it has the advantage of being rapid, reproducible, feasible and cost efficient. RPS6KB1 is a key factor in protein synthesis and has been found to be involved in a variety of human diseases ranging from diabetes, obesity, hemangioma to cancer [23–26]. A better understanding of RPS6KB1 in various pathological conditions could promote the development of strategies of diagnosis, prognosis and treatment schedules. In the current study, we explored the value of RPS6KB1 in predicting the 5-year OS for NSCLC patients. IHC staining revealed the up-regulated expression of total RPS6KB1 and its phosphorylated protein p-RPS6KB1 in primary NSCLC clinical samples, suggesting the potential role of RPS6KB1 in NSCLC. Similar results were also found in neuroendocrine tumor [27], breast carcinoma [28] and pancreatic ductal adenocarcinoma [29].

Further clinicopathologic analysis showed that only the high phosphorylation of RPS6KB1 was associated with the regional lymph node involvement and clinical progression in NSCLC patients. There was no significant difference in total RPS6KB1 level in terms of gender, age, tumor histological subtypes or TNM stage. It is probable that only the excessive phosphorylation of RPS6KB1, rather than the total protein overexpression, facilitates the rapid development of NSCLC cases. The unphosphorylated RPS6KB1, which is contained within the detected total RPS6KB1, is likely to be nonfunctional. In consistent with our results, overexpression of total RPS6KB1 showed no significant association with clinical variables in gastric cancer; but the level of p-RPS6KB1 revealed a strong correlation with tumor differentiation, depth of invasion and TNM stage [30]. Observations in esophageal squamous cell carcinoma that p-RPS6KB1 had a robustly positive expression in tumor tissues and closely related to the regional lymph node metastasis and advanced clinical stage provided more evidence that p-RPS6KB1 is probably the activated form of RPS6KB1 to exert its pathophysiological function in several kinds of tumors, including NSCLC [31].

Moreover, only the hyperphosphorylation of RPS6KB1 distinctly correlated with a worse 5-year OS in NSCLC patients after surgical resection, but the prognostic difference between the low and high RPS6KB1 expression group did not reach a statistical significance. This finding is a further clue that only RPS6KB1 phosphorylation is functional. Subsequent univariate analysis found p-RPS6KB1, along with clinical factors of tumor histological differentiation and TNM stage, also greatly associated with the median survival time in NSCLC patients, and the multivariate Cox regression analysis confirmed its independently prognostic predicting value. Based on these findings, we might be able to draw a conclusion that RPS6KB1 phosphorylation is a prognostic marker for NSCLC patients. Previous studies on astrocytomas [32], gastric cancer [30] and esophageal squamous cell carcinoma [33] also identified the phosphorylated RPS6KB1, but not total RPS6KB1, as a novel unfavorable prognosis indicator. However, there are conflicting results as well. In pancreatic ductal adenocarcinoma, neither RPS6KB1 nor p-RPS6KB1 was prognostic [34]. In spite of the detected rate of p-RPS6KB1 in kidney cancer was as high as 92% (60/65), no prognostic value was found [35]. We presume that these contradictory findings perhaps due to the heterogeneity in different kinds of tumors. In a wide variety of cancers, RPS6KB1 is regulated by Akt/mTOR signaling pathway [36, 37]. However, the network regulating tumor biological behaviors is exceedingly complicated, andRPS6KB1 could also work via other signaling pathways, such as miR-128/ HIF-1α/PKM2 [38]. So the negative effect of RPS6KB1 in pancreatic ductal adenocarcinoma and kidney cancer might not weaken the crucial role of RPS6KB1 in NSCLC.

In order to clarify the underlying mechanism of RPS6KB1 in NSCLC, the specific RPS6KB1 phosphorylation inhibitor LY2584702 was adopted to observe the NSCLC cells biological activities with RPS6KB1 dephosphorylation. A549 and SK-MES-1 were representative pulmonary adenocarcinoma and squamous carcinoma cell lines, which are the principal subtypes of NSCLC. As expected, loss of p-RPS6KB1, together with the corresponding downregulation of p-S6, suppressed the proliferation of NSCLC cell lines *in vitro*. Cell cycle redistribution, especially G0-G1 arrest to block cell division, might be the primary mechanism. Accordingly, cells in S and G2-M phases decreased simultaneously. As mentioned above, RPS6KB1 is prominent in protein synthesis, while most of the essential proteins regulating cell proliferations are produced in the S phase. So it is easy to understand the synchronous RPS6KB1 dephosphorylation and the reduction of cells in S phase, both in A549 and SK-MES-1. Moreover, apoptotic cells increased as well with LY2584702 treatment in A549, which perhaps another way to prevent cells proliferation. However, RPS6KB1 dephosphorylation had no demonstrable effect on SK-MES-1 apoptosis. These results indicated that there might be different molecular pathogenesis between the lung adenocarcinoma and squamous carcinoma. Similarly, the boswellic acid analog BA145 suppressed the T389 phosphorylation of RPS6KB1 in pancreatic cancer, leading to cell cycles distribution and inducing cell apoptosis [39], which is consistent with our findings.

Although data in our study included most clinicopathologic characteristics of NSCLC patients, due to the inherent nature of retrospective study, we could not bring all the recognized prognostic factors into our analysis. More studies with a larger sample size from multicenter and more in-depth molecular biological research are required to. Nevertheless, our study clarifies that RPS6KB1 is hyperphosphorylated in NSCLC, rather than just the total protein overexpressing. The overactivated RPS6KB1 predicts worse prognosis of NSCLC patients, and this might be attributed to promoting cells proliferation by regulating cell cycle distribution.
